# Studies on pollen micro-morphology, pollen storage methods, and cross-compatibility among grape (*Vitis* spp.) genotypes

**DOI:** 10.3389/fpls.2024.1353808

**Published:** 2024-02-21

**Authors:** Prabhanjan Rane, Madhubala Thakre, Mahendra Kumar Verma, Chavlesh Kumar, Jai Prakash, Vartika Srivastava, Shashank P. R., Niranjana Murukan, Gautam Chawla, Pranab Kumar Mandal, Harshit Kumar, Amol K. Jadhav, Eldho Varghese, Vishaw Bandhu Patel, Sanjay Kumar Singh

**Affiliations:** ^1^ Division of Fruits and Horticultural Technology, Indian Council of Agricultural Research (ICAR)-Indian Agricultural Research Institute, New Delhi, India; ^2^ ICAR-Central Institute of Temperate Horticulture, Srinagar, India; ^3^ Division of Germplasm Conservation, ICAR-National Bureau of Plant Genetic Resources, New Delhi, India; ^4^ Division of Entomology, ICAR-Indian Agricultural Research Institute, New Delhi, India; ^5^ Division of Genetics, ICAR-Indian Agricultural Research Institute, New Delhi, India; ^6^ Division of Nematology, ICAR-Indian Agricultural Research Institute, New Delhi, India; ^7^ ICAR-National Institute for Plant Biotechnology, New Delhi, India; ^8^ Fishery Resources Assessment, Economics & Extension Division, ICAR-Central Marine Fisheries Research Institute, Kochi, India; ^9^ Indian Council of Agricultural Research, New Delhi, India

**Keywords:** cross-compatibility, pollen germination, pollen micro-morphology, pollen viability, *Vitis parviflora* Roxb., *Vitis* spp.

## Abstract

The knowledge of pollen morphology, suitable storage condition, and species compatibility is vital for a successful grapevine improvement programme. Ten grape genotypes from three different species, *viz.*, *Vitis vinifera* L., *Vitis parviflora* Roxb., and *Vitis champini* Planc., were studied for their pollen structure and pollen storage with the objective of determining their utilization in grape rootstock improvement programs. Pollen morphology was examined through the use of a scanning electron microscope (SEM). The viability of the pollen was assessed using 2,3,5-triphenyltetrazolium chloride (TTC). *In vitro* pollen germination was investigated using the semi-solid medium with 10 % sucrose, 100 mg/L boric acid, and 300 mg/L calcium nitrate. The results revealed variations in pollen micro-morphology in 10 genotypes, with distinct pollen dimensions, shapes, and exine ornamentation. However, species-wise, no clear difference was found for these parameters. Pollen of *V. parviflora* Roxb. and Dogridge was acolporated and did not germinate. The remaining eight genotypes exhibited tricolporated pollen and showed satisfactory *in vitro* pollen germination. Storage temperature and duration interactions showed that, at room temperature, pollen of most of the grape genotypes can be stored for up to 1 day only with an acceptable pollen germination rate (>30 %). However, storage for up to 7 days was successfully achieved at 4 °C, except for ‘Pearl of Csaba’. The most effective storage conditions were found to be at −20 °C and −196 °C (in liquid N_2_), enabling pollen storage for a period of up to 30 days, and can be used for pollination to overcome the challenge of asynchronous flowering. Four interspecific combinations were studied for their compatibility, among which *V. parviflora* Roxb. × *V. vinifera* L. (Pusa Navrang) and *V. parviflora* Roxb. × *V. champini* Planc. (Salt Creek) showed high cross-compatibility, offering their potential use for grape rootstock breeding. However, *V. parviflora* Roxb. × *V. vinifera* L. (Male Hybrid) recorded the lowest compatibility index among studied crosses. In the case of self-pollinated flowers from *V. parviflora* Roxb. and *V. parviflora* Roxb. × *V. champini* Planc. (Dogridge), pollen failed to germinate on the stigma due to male sterility caused by acolporated pollen. As a result, the flowers of these genotypes functioned as females, which means they are ideal female parents for grape breeding without the need for the tedious process of emasculation.

## Introduction

The grapevine, scientifically known as *Vitis vinifera* L., is a member of the Vitaceae family, which includes approximately 60 inter-fertile wild *Vitis* species found in Asia, North America, and Europe. The European grape (*V. vinifera* L.) originated primarily in the Caucasus region between the Caspian Sea and the Black Sea. In contrast, the American grapes, including *Vitis labrusca*, other species of *Euvitis*, and *Muscadinia*, originated in North America (Winkler, 1965). The Himalayan region of India may be considered a secondary center of origin (Sankaran and Dinesh, 2020). Grape cultivation stands as a highly profitable venture in India, particularly thriving in subtropical and tropical zones. In the fiscal year 2022–23, India emerged as a major exporter, dispatching 267,950.39 metric tons of fresh grapes with a total value of Rs. 2,543.42 crores or 313.70 USD million (Anonymous, 2023).

Pollen plays a critical role in grape production, influencing fruit setting volume. The fertility of grape pollen depends on its viability and germination potential (Lombardo et al., 1978). In the context of breeding, palynological studies are mandatory before designing rootstock and scion hybridization programs. Under palynological research, cultivated plants are characterized based on pollen grain micro-morphology, which is a vital indicator for taxonomic and fertility purposes, and is frequently used to address theoretical and applied aspects of production. Inconsistent yields in specific grapevine types might be linked to different pollen types like bicolporate, acolporate, shriveled, or collapsed forms (Caporali et al., 2003; Abreu et al., 2006). At the same time, grape cultivars with a pollen germination rate equal to or greater than 30% can be employed as effective pollinators (Fidan, 1975), a significant factor in cultivar selection for grape vineyards. However, palynological studies can be used to study the complicated fertilization biology of grapevines (Sabir, 2015). Variation in pollen morphology is captured by palynological studies performed by Cargnello et al. (1980); Dzyuba et al. (2006), and Gallardo et al. (2009). Usually, pollen of *V. vinifera* L. is tricolporated, i.e., the presence of three furrows, with a prolate to triangular shape (Abreu et al., 2006; Alva et al., 2015). In addition to this, pollen storage enables hybridization between desired parent genotypes cultivated in different geographic locations, especially when their flowering is asynchronous within a region (Sharafi and Bahmani, 2011; Mesnoua et al., 2018).

Initial grape rootstock improvement used the pure form of *V. riparia* Michx. and *V. rupestris* Scheele. as rootstock and they were easy to root and graft compatible with *V. vinifera* L. However, in some cases, like *Vitis berlandieri* Planchon., rooting and grafting were difficult. Hence, later on, hybridization of two different species was attempted for grape rootstock breeding, *viz.*, *V. berlandieri* × *V. rupestris* (Bavaresco et al., 2015) and *V. riparia* × *V. rupestris* (Pavloušek, 2015).

*Vitis champini* cv. Dogridge dominates the Indian grape industry as the primary commercially favored rootstock due to its benefits in terms of moisture stress tolerance, chloride exclusion, and enhanced scion vigor (Somkuwar et al., 2012). However, relying solely on Dogridge poses a significant risk to the grape industry, as its performance can be variable under different climatic conditions, e.g., Dogridge can induce higher vigor in the scion under tropical and subtropical climates, reducing bud fruitfulness (Satisha et al., 2010). Various *Vitis* species, including *V. champinii*, *V. berlandierii*, *V. rupestris*, *Vitis longii*, and *Vitis parviflora*, have shown the ability to produce biochemical compounds that influence the physiology, root structure, development, and distribution of the scion (Somkuwar et al., 2012), which can be utilized for rootstock improvement.

Harnessing the vast pool of genetic diversity remains largely untapped in grape rootstock breeding specifically under Indian conditions. Popular grape rootstocks have a narrow genetic base derived from a few American grape species (Riaz et al., 2019). *V. parviflora* Roxb., a Himalayan wild grape species, possesses desirable traits such as moderate vigor, drought resistance, and multiple disease resistance, making it a promising candidate as a rootstock (Singh et al., 2022). Combining the traits of this wild species with those of other grape species may result in rootstock tolerant to abiotic and biotic stresses with wider adaptability. However, comprehensive knowledge of its combining ability in terms of pollen–pistil interaction, fruit set percentage, seed setting percentage, and other factors is essential for grape improvement work. In light of this context, the study involved an examination of the pollen micro-morphology of different grape genotypes. The subsequent sections focused on examining pollen storage, specifically targeting the challenge posed by asynchronous flowering. Additionally, the study explored the compatibility of *V. parviflora* with other *Vitis* species, aiming to strengthen the grape rootstock breeding program by leveraging genetic diversity for enhanced adaptability and stress resistance.

## Materials and methods

### Plant material

The experiment was carried out at a vineyard (28°38′37.8″N, 77°09′27.8″E) of the Division of Fruits and Horticultural Technology, ICAR-Indian Agricultural Research Institute, New Delhi, in 2023. The plant materials for the study were comprised of 10 grape genotypes belonging to three species, i.e., *V. vinifera* L., *V. champini* Planc., and *V. parviflora* Roxb., and were selected for pollen micro-morphology and storage studies (**Table 1**). All vines were own-rooted, 10 years old, and trained on a bower system. For cross-compatibility study, Salt Creek (*V. champini* Planc.), Male Hybrid (*V. vinifera* L.), Pusa Navrang (*V. vinifera* L.), and Dogridge (*V. champini* Planc.) were used as male parents and *V. parviflora* Roxb. as a female parent.

**Table 1:** Description of *Vitis* genotypes.

I. *Vitis vinifera* L.
**1. Pearl of Csaba (POC):** Earliest variety in North India. Used in hybridization programs for inducing earliness and sweetness (Chadha and Shikhamany, 1999).
**2. Perlette (PER):** Hybrid of Scolokertek Hiralynoje × Sultania marble. The early variety of North India ripens by the third week of May to third week of June depending upon the region of cultivation (Chadha and Shikhamany, 1999).
**3. Early Perlette Selection (EPS):** The early genotype was selected from Perlette from IARI. It is earlier in flowering than Pearl of Csaba and Perlette.
**4. Beauty Seedless (BS):** It is an introduction from California, USA. An early ripening, colored, seedless, prolific bearing grape variety (Chadha and Shikhamany, 1999).
**5. Pusa Navrang (PN):** It is a cross between Madeleine Angevine and Ruby Red. Teinturier grape hybrid with early ripening and basal bearing. Vines are resistant to anthracnose (Chadha and Shikhamany, 1999).
**6. Flame Seedless (FS):** Hybrid introduced from USA. Early maturing, higher yield with better fruit quality, loose bunches, and tolerance to rain (Chadha and Shikhamany, 1999).
**7. Male Hybrid (MH):** It is hybrid rootstock developed from Banqui Abyad × Victory (74-9) with functional male flowers.
II. *Vitis champini* Planc.
**1. Dogridge (DR):** Rootstock, resistant to nematodes and tolerant to salinity. Vines have very vigorous spreading and prostrate habit. Recommended for use in lighter and less fertile sandy soils as well as drought-prone areas (Chadha and Shikhamany, 1999).
**2. Salt Creek (SC) *Syn*. Ramsay:** Rootstock, resistant to nematodes and tolerant to salinity. It imparts great vigor to scions but not as much as Dogridge can (Chadha and Shikhamany, 1999).
III. *Vitis parviflora* Roxb.
**1. VP:** It is indigenous to the Himalayan region in India. It is used in the breeding program to induce disease-pest resistance and tolerance to drought (Chadha and Shikhamany, 1999).

### Pollen collection

Inflorescences expected to bloom within 7–8 days were covered with perforated butter paper bags. Pollen was collected from bagged panicles of three vines of each genotype between 7 am to 9 am. These panicles were monitored regularly and excised when they reached “EL 23 Full flowering: 50 % flower hoods fallen” according to the Eichhorn and Lorenz (E-L) system (Lorenz et al., 1995). Subsequently, the collected clusters were transferred to the laboratory and dried for 24 hours at room temperature. Collected floral debris was passed through sieves to obtain pollen and transferred to 1.5ml vials by brush (Rane et al., 2023).

### Scanning electron microscopy of pollen

Dehydration of pollen was performed by placing vials with their lids open in the silica gel desiccator at 4 °C until analysis with proper labeling to maintain their identity and avoid contamination (Lukšić et al., 2022). After dehydration, the small quantities of pollen were mounted for examination using a fine brush on aluminum stubs that were coated with double-sided transparent tape. Subsequently, a 0.02-µm-thick gold layer was applied using a sputter coater (EMITECH SC7620 mini sputter coater). At each stage, contamination of samples by pollen from different genotypes was avoided. The samples were examined at 10 kV using a scanning electron microscope (TESCAN VEGA3). Measurements of 30 pollen grains from each cultivar were taken and recorded as data for three separate replicates per genotype, with each replicate consisting of 10 pollen grains. These observations were made at a magnification of ×3,000 for whole grain and ×15,000 for exine ornamentation. The pollen traits were, namely, number of apertures, length of polar axis (P), length of equatorial axis (E), P/E ratio, length and width of colpus, mesocolpium width, and exine surface ornamentation. Shapes of pollen were categorized according to Erdtman (1971).

### Pollen storage

The pollen samples for storage study were prepared separately. For this purpose, pollen was stored in sealed vials with proper labeling. Pollen of Early Perlette Selection (EPS), Pearl of Csaba (POC), PER, Pusa Navrang (PN), Beauty Seedless (BS), Flame Seedless (FS), Male Hybrid (MH), and Salt Creek (SC) was stored under four different storage conditions, *viz.*, at room temperature, 4 °C, −20 °C, and −196 °C (in liquid N_2_). *In vitro* pollen germination and viability were tested after the 0th day (just after fresh pollen collection), 1st day, 3rd day, 5th day, 7th day, 15th day, and 30th day of storage under each storage condition. The samples were stored in multiple sets of vials for each genotype to minimize the stress linked to thawing.

### Pollen viability

The determination of pollen viability was performed by using 1% of 2,3,5-triphenyltetrazolium chloride (TTC) diluted in a 50 % sucrose solution (Shivanna, 2003 modified). One drop of solution was placed on a labeled slide, and stored pollen grains were spread with a brush on the slide and covered with a coverslip on the top. The slides were placed in a Petri dish and placed in the dark at 35°C with 50 % relative humidity (RH) for 6 hours. Pollen viability counts were made under a light microscope (OLYMPUS CX33, Tokyo, Japan). Pollen grains were considered viable if they were stained with orange or bright red color. Three randomly chosen microscopic areas (each with a minimum of 100 pollen grains) were used to count the pollen grains on each slide.

### *In vitro* pollen germination

*In vitro* pollen germination was observed on standardized semi-solid medium containing 10 % sucrose + 100 mg/L boric acid + 300 mg/L calcium nitrate and agar 0.6%, at pH 5.85 (**Supplementary Table 1**). A small rectangular piece of medium was removed by a scalpel and placed on a labeled slide. Pollen grains were dusted uniformly on the surface of the medium with a fine brush. Slides were placed in a Petri dish with damp filter paper and covered with a lid to avoid drying of the medium and maintain humidity. The Petri dishes were placed in a dark environment for incubation. The growth of pollen tubes on the medium was observed under a light microscope (OLYMPUS CX33) after 24 hours of incubation. Pollen grain was considered germinated if the length of the tube exceeded the pollen (Nepi and Franchi, 2000). The germination rate was calculated by counting three fields per sample, each comprising 100 pollen grains.

### Cross-compatibility study

#### Crossing

The cross-combinations, *V. parviflora* Roxb. × *V. vinifera* L. (Pusa Navrang), *V. parviflora* Roxb. × *V. vinifera* L. (Male Hybrid), *V. parviflora* Roxb. × *V. champini* Planc. (Salt Creek), *V. parviflora* Roxb. × *V. champini* Planc. (Dogridge), and selfed *V. parviflora* Roxb. were considered for crossing and cross-compatibility study. Pollen of the male parents [PN, MH, SC, and DR] was collected freshly from previously bagged clusters in the morning by shaking the clusters on Petri dishes when almost 50 % of the flowers were opened. After pollination, the whole cluster was immediately covered with a butter paper bag to avoid unwanted cross-pollination and marked with tags with details of parents and the date of pollination.

#### Fixation of crossed pistils

After 24 hours of pollination, flowers were fixed in a formalin-aceto-alcohol (FAA) solution with a composition of 37% formaldehyde (10 ml), 95 % ethyl alcohol (50 ml), glacial acetic acid (5 ml), and water (35 ml) as specified by García-Breijo et al., (2020). After fixation, flowers were immediately stored in the refrigerator at 4 °C until analysis. The remaining crossed clusters were kept for fruit set observation and were allowed to grow similarly to other clusters on the vine in open sunlight throughout berry development and maturation (Sabir, 2015).

#### Fluorescence microscopy for *in vivo* pollen tube growth

The flowers that had been fixed were longitudinally sectioned to 20 µm using a freezing microtome (Thermo Scientific Microm HM550, Waltham, MA, USA). These sections were then subjected to staining with aniline blue (0.1% in phosphate-buffered saline (PBS) buffer) for 15 minutes. Following staining, the sections were rinsed with distilled water and affixed to a microscope slide using mounting medium. Mounted sections were examined using a fluorescence microscope (García-Breijo et al., 2020). For fluorescence microscopy, a Nikon H600L fluorescence microscope with a DAPI cube (excitation filter 362–396 nm, dichroic mirror 415 nm, barrier filter 432–482 nm) was used. A total of 25 pistils were observed for each cross-combination.

#### Fruit set, number of seeds per berry, and compatibility index

Crossability indices like final berry retention (fruit set %), the number of seeds per berry, and compatibility index (Patil et al., 2013) were calculated from observed data. Fully ripened berries were harvested and cut to count the number of seeds per fruit. The number of seeds was calculated from 150 berries from each combination. The compatibility index was determined using the ratio of the number of fully mature seeds harvested to the total count of flowers that could be pollinated (Wang et al., 2022).

### Statistical analysis

Analysis of variance (ANOVA) was performed using PROG GLM of the SAS software package, version 9.4 (SAS Institute, Cary, NC, USA). Pollen dimension and shape-related parameters were analyzed through one-way ANOVA. Further, a two-way ANOVA was performed for each genotype separately, with storage temperature and duration as the factors, to study their impact on pollen germination (%) and viability. Tukey's Honest Significant Difference (HSD) test was used to identify the pairwise significant differences among the various factors and their interactions. Data were transformed using square root transformation of the form (x + 0.5), {i.e.√(x + 0.5)} for the parameters like colpus length, colpus width, mesocolpium width, pollen viability, and fruit set (%), and arcsine transformation was used for pollen germination (%) to make it amenable for ANOVA. A p < 0.05 was considered statistically significant.

## Results

### Type of flower and its relationship with pollen micro-morphology

**Figure 1:**
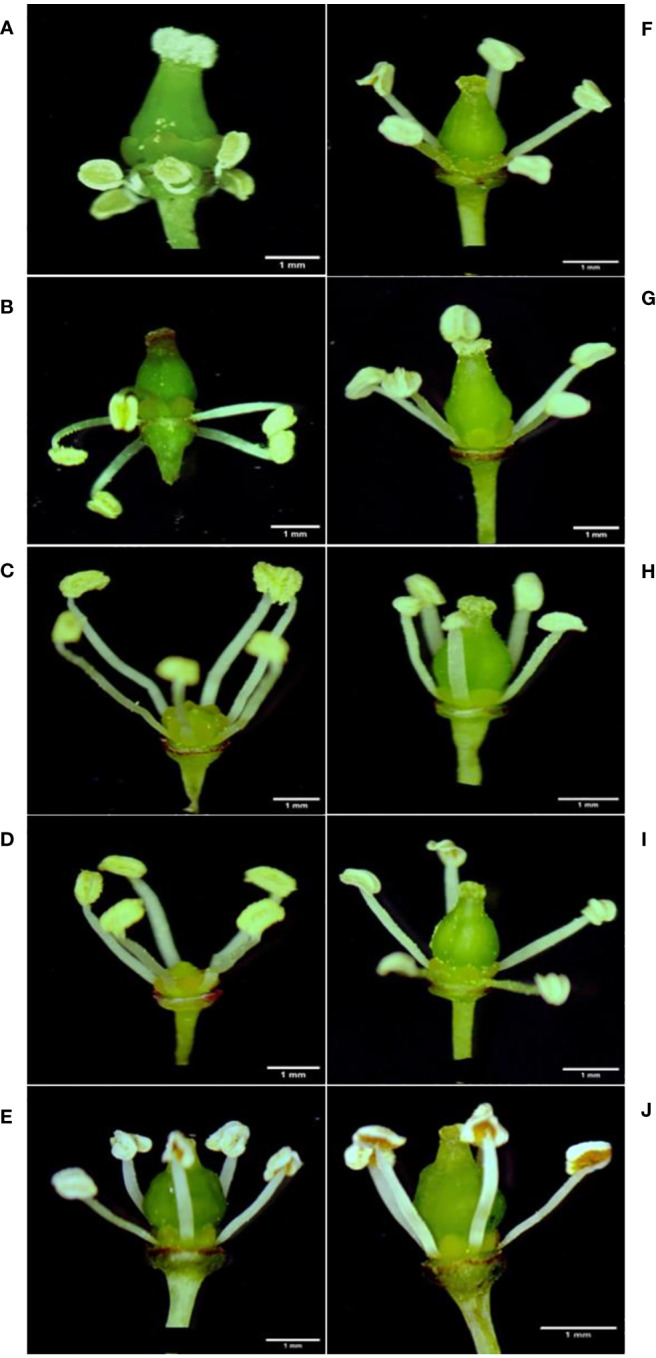
Types of flowers in grape genotypes. Flowers with reflexed stamens in Dogridge **(A)**, *Vitis parviflora* Roxb. **(B)** Functionally staminate flower in Male hybrid **(C)** and Salt Creek **(D)**. Hermaphrodite flower in Early Perlette Selection **(E)**, Pearl of Csaba **(F)**, Pusa Navrang **(G)**, Perlette **(H)**, Flame Seedless **(I)**, and Beauty Seedless **(J)**.

**Figure 2:**
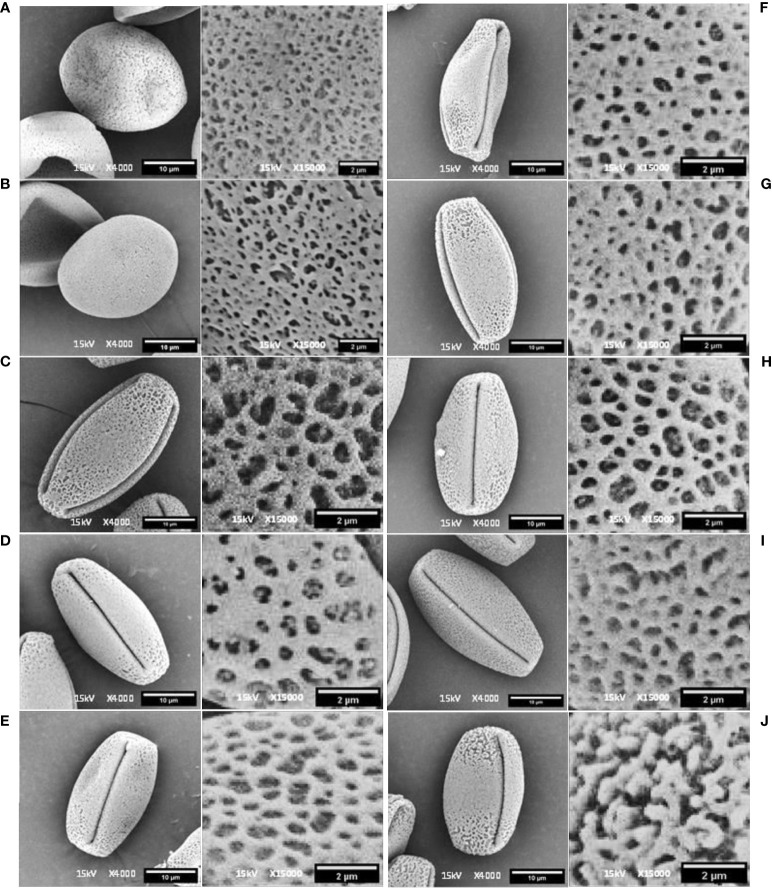
Scanning electron microscopy image of pollen in equatorial view and detailed exine pollen surface in Dogridge **(A)**, *Vitis parviflora* Roxb. **(B)**, Male hybrid **(C)**, Salt Creek **(D)**, Early Perlette Selection **(E)**, Pearl of Csaba **(F)**, Pusa Navrang **(G)**, Perlette **(H)**, Flame Seedless **(I)**, and Beauty Seedless **(J)**.

Among the 10 *Vitis* genotypes investigated in this study, EPS, PN, POC, PER, BS, and FS from *V. vinifera* L. were identified as hermaphrodites, exhibiting well-developed stamens and pistils. However, two genotypes, MH from *V. vinifera* L. and SC from *V. champini* Planch., were functionally male, having a rudimentary ovary and well-developed stamens (**Figure 1**). VP and DR (*V. champini* Planc.) had well-developed pistils and reflexed stamens. The pollen of genotypes with reflexed stamens (DR and VP) exhibited inaperturate or acolporate pollen, which was subprolate (P/E ratio: 1.14–1.33) in shape (**Figures 2A, B** and **Table 2**). However, the remaining genotypes were tricolporate and prolate (P/E ratio: 1.33–2.00) in shape (**Figures 2C–J** and **Table 2**). The EPS and MH genotypes had reticulate exine ornamentation, while BS had reticulate-striate exine ornamentation. The remaining genotypes, i.e., PN, PER, POC, FS, DR, and VP, possess foveolate-perforate exine ornamentation.

**Table 2:** Pollen micro-morphology details of various grape genotypes observed under SEM.

Genotype	Polar axis (P, µm)	Equatorial axis (E, µm)	P/E ratio	Shape	Colpus length (µm)	Colpus width (µm)	Mesocolpium width (µm)	Exine surface ornamentation
EPS	28.600^ab^	15.509^cd^	1.864^a^	Prolate*	24.754^b^	0.506^bc^	12.001^ab^	Reticulate
PN	26.097^bc^	14.786^cd^	1.769^a^	Prolate*	22.668^bc^	0.809^a^	10.856^bc^	Foveolate-perforate
POC	27.692^ab^	14.199^d^	1.962^a^	Prolate*	24.163^bc^	0.509^bc^	9.886^c^	Foveolate-perforate
PER	27.571^ab^	15.515^cd^	1.786^a^	Prolate*	23.379^bc^	0.653^ab^	11.503^bc^	Foveolate-perforate
BS	25.802^bc^	14.090^d^	1.842^a^	Prolate*	22.119^c^	0.542^bc^	10.944^bc^	Reticulate-Striate
FS	25.546^bc^	14.148^d^	1.827^a^	Prolate*	24.574^b^	0.533^bc^	11.187^bc^	Foveolate-perforate
MH	31.181^a^	16.706^bc^	1.867^a^	Prolate*	28.027^a^	0.378^c^	13.599^a^	Reticulate
SC	28.373^ab^	15.039^cd^	1.887^a^	Prolate*	23.840^bc^	0.509^bc^	11.428^bc^	Foveolate-perforate
DG	26.115^bc^	18.754^a^	1.264^b^	Sub prolate**	0^d^	0^d^	0^d^	Foveolate-perforate
VP	23.524^c^	18.754^ab^	1.255^b^	Sub prolate**	0^d^	0^d^	0^d^	Foveolate-perforate
LSD (p ≤ 0.05)	2.097	1.39	0.148		(0.137)	(0.006)	(0.525)	

EPS, Early Perlette Selection; PN, Pusa Navrang; POC, Pearl of Csaba; PER, Perlette; BS, Beauty Seedless; FS, Flame Seedless; MH, Male Hybrid; SC, Salt Creek; VP, *Vitis parviflora* Roxb.; DG, Dogridge; SEM, scanning electron microscopy.

According to Erdtman (1971).

*P/E ratio = 1.33-2.00 (prolate).

**P/E ratio = 1.14:1.33 (subprolate).

Least significant difference (LSD) values in parentheses indicate LSD for transformed data. The same superscript indicates that the values do not differ significantly.

The study of pollen dimensions (**Table 2**) showed that the length of the polar axis ranged from 23.524 µm to 31.181 µm and that the length of the equatorial axis ranged from 14.090 µm to 20.657 µm among the 10 grape genotypes. The average values for the P/E ratio varied between 1.255 and 1.962. The P/E ratio did not differ significantly in the tricolporate type of pollen. However, acolporate or inaperturate pollen had the lowest P/E ratio. The minimum colpus length (22.119 µm) was recorded in genotype BS. However, PN had the maximum colpus width (0.809 µm), which differed non-significantly from PER (0.653 µm). However, the minimum colpus width (0.378 µm) was observed in MH, which was statistically non-significant with EPS, POS, BS, FS, and SC. Considerable variation was also recorded in the mesocolpium width of all studied genotypes ranging from 9.886 µm to 13.599 µm (**Table 2**).

### Characteristics of fresh pollen

**Figure 3:**
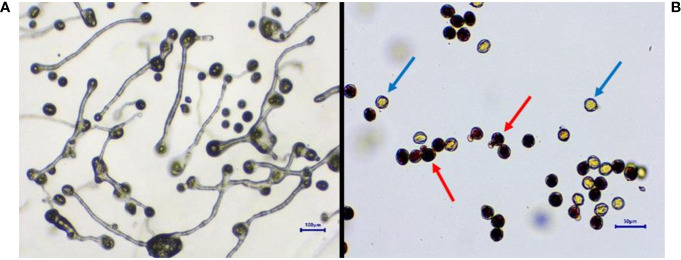
Determination of pollen germination and viability. *In vitro* pollen germination. **(A)** Pollen viability determination by TTC. **(B)** Blue arrow, non-viable; red arrow, viable. TTC, 2,3,5-triphenyltetrazolium chloride.

**Figure 4:**
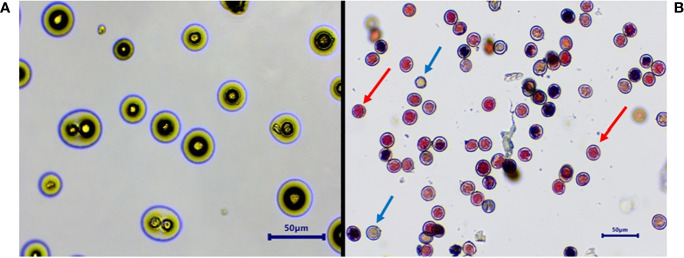
Inaperturate pollen of *Vitis parviflora* Roxb. showing no pollen germination **(A)** and showing pollen viability by staining with red color **(B)**. Blue arrow, non-viable; red arrow, viable.

In all studied *Vitis* genotypes, maximum germination and viability were recorded in fresh pollen (**Figure 3**). The germination of fresh pollen ranged from 40.4% (POC) to 90.4 % (FS) (**Table 3**), while pollen viability rate ranged from 40% (POC) to 95 % (FS). Notably, there was a higher degree of variation observed among the genotypes concerning both pollen viability and germination. However, pollen of DR and VP were inaperturate and did not germinate *in vitro*, but they did show viability during staining by TTC (**Figure 4**). The pollen of these two genotypes was not studied further for storage studies.

**Table 3:** Effect of storage temperature and duration on the *in vitro* pollen germination of different *Vitis* genotypes.

Genotype	Temperature	0 day of storage	1^st^ day of storage	3^rd^ day of storage	5^th^ day of storage	7^th^ day of storage	15^th^ day of storage	30^th^ day of storage
Early Perlette Selection	RT 4°C –20°C –196°C	74.9^a^	43.2^bc^ 71.2^a^ 70.3^a^ 71.5^a^	31.7^cd^ 71.6^a^ 69.9^a^ 72.2^a^	24.1^de^ 71.3^a^ 69.6^a^ 70.2^a^	10.3^e^ 69.2^a^ 68.2^a^ 70.5^a^	0^f^ 64.2^a^ 66.5^a^ 69.6^a^	0^f^ 61.9^ab^ 64.4^a^ 70.3^a^
Pusa Navrang	RT 4°C –20°C –196°C	61.3^a^	39^d^ 59.3^ab^ 60.5^a^ 58.2^ab^	16.3^e^ 59.8^ab^ 60.5^a^ 58.8^ab^	0^f^ 57.7^ab^ 58.9^ab^ 59^ab^	0^f^ 54.5^abc^ 56.7^ab^ 58.8^ab^	0^f^ 48.8^bcd^ 53.7^abc^ 59.4^ab^	0^f^ 44.6^cd^ 50.3^abc^ 58.9^ab^
Pearl of Csaba	RT 4°C –20°C –196°C	40.4^a^	31.1^abc^ 36.2^ab^ 39.2^a^ 39.8^a^	3.5^e^ 34.2^ab^ 39.2^a^ 39.9^a^	0^f^ 29.9^abc^ 36.3^ab^ 38.7^a^	0^f^ 25.1^bc^ 32.1^abc^ 39.2^a^	0^f^ 22.2^cd^ 30.3^abc^ 38.5^a^	0^f^ 12.6^d^ 22.6^c^ 38.3^a^
Perlette	RT 4°C –20°C –196°C	64.5^a^	56.7^abc^ 62.6^ab^ 63.5^a^ 63.9^a^	47.1^cd^ 62^ab^ 63.6^a^ 63.3^ab^	31.8^e^ 60.5^ab^ 62.5^ab^ 63.2^ab^	0.7^f^ 57.3^abc^ 60.5^ab^ 63.4^ab^	0^f^ 51.5^bcd^ 60.8^ab^ 63.3^ab^	0^f^ 42.5^de^ 60^ab^ 62.2^ab^
Beauty Seedless	RT 4°C –20°C –196°C	68.3^a^	47.8^c^ 63.5^ab^ 66.2^a^ 65.8^a^	28.8^d^ 63.6^ab^ 66.3^a^ 66.4^a^	13.4^e^ 61.3^ab^ 63.5^ab^ 65.5^a^	7^e^ 60.5^ab^ 63.8^ab^ 65.3^a^	0^f^ 58a^bc^ 63.5^ab^ 66.4^a^	0^f^ 53.3^bc^ 62.9^ab^ 66.1^a^
Flame Seedless	RT 4°C –20°C –196°C	90.4^a^	71.1^cde^ 88.5^ab^ 89.2^ab^ 88.6^ab^	68.6^de^ 86.5^ab^ 89.3^ab^ 87.2^ab^	59.4^ef^ 83.5^ab^ 87.8^ab^ 87.4^ab^	51.6^f^ 81.4^abc^ 86.8^ab^ 86.5^ab^	2.9^g^ 79.6^bcd^ 84.9^ab^ 87.2^ab^	0^g^ 70.5^cde^ 82.5^abc^ 86.5^ab^
Male Hybrid	RT 4°C –20°C –196°C	71.7^a^	44.1^d^ 69.3^ab^ 70.6^ab^ 71.1^a^	41^de^ 69.3^ab^ 70.7^ab^ 68^ab^	32.3^ef^ 68.7^ab^ 70.4^ab^ 67^ab^	26.7^f^ 66.8^ab^ 69.8^ab^ 68.1^ab^	0^g^ 55.9^c^ 62.2^abc^ 68.1^ab^	0^g^ 51.3^cd^ 60.4^bc^ 67.3^ab^
Salt Creek	RT 4°C –20°C –196°C	76.7^a^	68.1^abc^ 73.2^ab^ 74.3^a^ 72.5^ab^	56.7^de^ 73^ab^ 74. 2^a^ 72.4^ab^	49.2^e^ 71.3^abc^ 73.6^ab^ 71.9^ab^	46.7^e^ 68.2^abc^ 73. 6^ab^ 72.6^ab^	0^f^ 64.3^bcd^ 72.1^ab^ 72^ab^	0^f^ 62^cd^ 70.9^abc^ 72.3^ab^

The same superscript indicates that the values do not differ significantly.

### Effect of storage temperature on the germination and viability of pollen

In this study, four different temperatures, *viz.*, room temperature, 4 °C, −20 °C, and −196 °C, were used to store pollen. For better understanding and to reduce the repetition, the results are discussed storage temperature-wise. At all the storage temperatures in every genotype, the maximum pollen germination and pollen viability was on the day of collection (**Tables 3**, **4**).

**Table 4:** Effect of storage temperature and duration on the pollen viability of different *Vitis* genotypes.

Genotype	Temperature	0^th^ day of storage	1^st^ day of storage	3^rd^ day of storage	5^th^ day of storage	7^th^ day of storage	15^th^ day of storage	30^th^ day of storage
Early Perlette Selection	RT 4°C –20°C –196°C	76.8^a^	46.2^c^ 73.3^ab^ 75.9^a^ 74.6^a^	33.2^d^ 73.3^ab^ 75.8^a^ 74.5^ab^	24.6^e^ 73.9^ab^ 74.9^a^ 74^ab^	15.6^f^ 72.9^ab^ 74.9^a^ 74^ab^	0^g^ 68.3^ab^ 74^ab^ 74.6^a^	0^g^ 64.6^b^ 73.5^ab^ 74.9^a^
Pusa Navrang	RT 4°C –20°C –196°C	66.6^a^	41.6^f^ 61.8^abc^ 64.3^ab^ 63.5^abc^	20.4^g^ 60.5^abd^ 63.2^abc^ 63.2^abc^	0^h^ 60^abcd^ 59.6^abcd^ 62.4^abc^	0^h^ 55.6^cde^ 57^bcd^ 62.3^abc^	0^h^ 53.1^de^ 55.5^cde^ 62.9^abc^	0^h^ 48.6^ef^ 53.3^de^ 62^abc^
Pearl of Csaba	RT 4°C –20°C –196°C	40^a^	36.6^ab^ 38.4^ab^ 38.9^a^ 40.3^a^	2.3^e^ 37.9^ab^ 39^a^ 41^a^	0^f^ 37.6^ab^ 38.3^ab^ 40.7^a^	0^f^ 37.2^ab^ 37.9^ab^ 39.4^a^	0^f^ 29.9^bc^ 34.1^abc^ 39.5^a^	0^f^ 18.2^d^ 26.6^c^ 39.8^a^
Perlette	RT 4°C –20°C –196°C	67.7^a^	57.5^abc^ 63.5^ab^ 63.5^ab^ 65.2^a^	41.6^d^ 63.5^ab^ 64^a^ 65.2^a^	4.3^e^ 62.7^ab^ 63.3^ab^ 64.2^a^	4.5^e^ 59.5^ab^ 63.9^a^ 64.9^a^	0^f^ 50.8^bcd^ 63.1^ab^ 64^a^	0^f^ 45.8^cd^ 63^ab^ 64^a^
Beauty Seedless	RT 4°C –20°C –196°C	72.5^a^	50.4^c^ 70.6^a^ 71.3^a^ 69.6^a^	29.7^d^ 68.1^a^ 70.2^a^ 69.1^a^	15.5^e^ 67.2^ab^ 70.2^a^ 70^a^	9.9^f^ 64.9^ab^ 70.6^a^ 69.6^a^	0^g^ 61.4^ab^ 68.8^a^ 70.2^a^	0^g^ 56.4^bc^ 65.6^ab^ 70.2^a^
Flame Seedless	RT 4°C –20°C –196°C	95^a^	75^fgh^ 90.5^a..d^ 91.1^abc^ 92.6^ab^	71.2^h^ 88.6^a..d^ 90.5^a..d^ 92.3^ab^	61.4^i^ 83.7^cde^ 88.3^a…e^ 93.1^ab^	53.9^j^ 82.7^def^ 87.6^a...e^ 91.6^abc^	4.8^k^ 80.6^efg^ 85.6^b..e^ 91.3^abc^	0^l^ 73.5^gh^ 83.5^cde^ 92.4^ab^
Male Hybrid	RT 4°C –20°C –196°C	75.6^a^	50.6^e^ 73.6^ab^ 73.8^ab^ 74.2^ab^	36.6^f^ 71^abc^ 73^ab^ 74.9^a^	34.2^f^ 70.7^abc^ 72.1^ab^ 73.7^ab^	30.6^f^ 69.3^abc^ 71.3^ab^ 73.1^ab^	0^g^ 61.6^cd^ 66.9^abcd^ 73.3^ab^	0^g^ 58.3^de^ 64.8^bcd^ 73.6^ab^
Salt Creek	RT 4°C –20°C –196°C	81.2^a^	70.2^a..d^ 77.3^ab^ 77.6^ab^ 74.3^a..d^	55.1^ef^ 74.6^a..d^ 77.4^ab^ 75.2^a..d^	50.8^f^ 73.6^a..d^ 77^ab^ 74.2^a..d^	47.7^f^ 69.7^bcd^ 76.4^ab^ 74.6^a..d^	0^g^ 65.2^cd^ 76.7^ab^ 72.3^a..d^	0^g^ 64.6^de^ 76^abc^ 73.5^a..d^

The same superscript indicates that the values do not differ significantly.

Pollen stored at room temperature recorded a sharp reduction in germination and viability rate over time in all the genotypes. At room temperature, pollen showed germination and viability extending up to the seventh day of storage. Beyond this period, it declined to zero in all grape genotypes except POC and PN. In the case of these two genotypes, storage for only 3 days was possible. However, the acceptable pollen germination percentage of >30% was found up to only the first day of storage in PN, POC, and BS; up to the third day in EPS; up to the fifth day in PER and MH; and a maximum of up to 7 days in FS and SC (**Tables 3**, **4**). FS and SC recorded pollen germination rates of 51.6 % and 46.7 %, respectively, even on the seventh day of storage, which is significantly higher than those of other studied genotypes under similar storage conditions. In all the genotypes, pollen germination was consistently the lowest when stored at room temperature, regardless of the duration. The decline in both pollen germination and viability occurred notably faster at room temperature compared to the other storage temperatures.

Pollen germination and pollen viability at 4 °C are presented in **Tables 3**, **4**. In all genotypes, pollen germination was more than acceptable, i.e., 30 % during 30 days of storage except in POC, which had it for only up to the third day of storage. In all the genotypes, pollen viability was significantly higher at 4 °C as compared to room temperature.

In all the genotypes, pollen germination percentage had a non-significant difference during the whole storage period at −20 °C and −196°C except in POC. Pollen stored at −196 °C maintained higher pollen germination compared to other storage conditions. However, in the case of pollen viability, the non-significant difference between pollen viability at different storage durations exists only at −196°C. The pollen storage at −20 °C had intermediate pollen viability with respect to pollen viability at −4°C and −196 °C.

### Cross-compatibility

#### *In vivo* pollen tube growth

**Figure 5:**
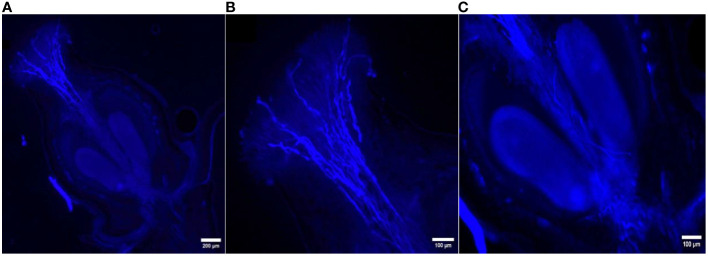
Longitudinal aniline blue-stained, fluorescent, frozen section (20 μm) of *Vitis parviflora* Roxb. × Pusa Navrang (*Vitis vinifera* L.) flowers. Pistil with visible abundant germinating pollen grains **(A)**. Pollen tubes penetrating transmission tissues of the style, with most of the pollen tubes reaching the base of the stylar canal. Magnified stigma with growing pollen tubes **(B)**. Pollen tube penetrating inside the ovule **(C)**.

**Figure 6:**
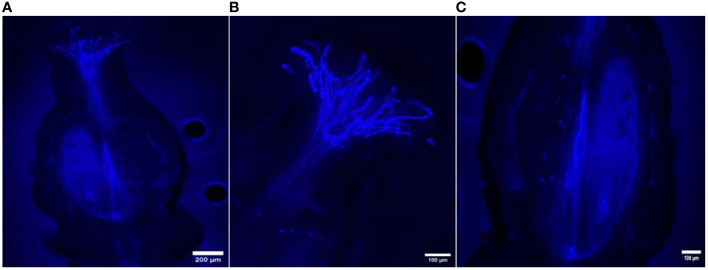
Longitudinal aniline blue-stained, fluorescent, frozen section (20 μm) of *Vitis parviflora* Roxb. × Salt Creek (*Vitis champini* Planc.) flowers. Pistil with visible abundant germinating pollen grains; pollen tubes penetrating the transmission tissues of the style, with most of the pollen tubes reaching the base of the stylar canal **(A)**. Stigma with growing pollen tubes **(B)**. Pollen tubes reaching the base of the ovary **(C)**.

**Figure 7:**
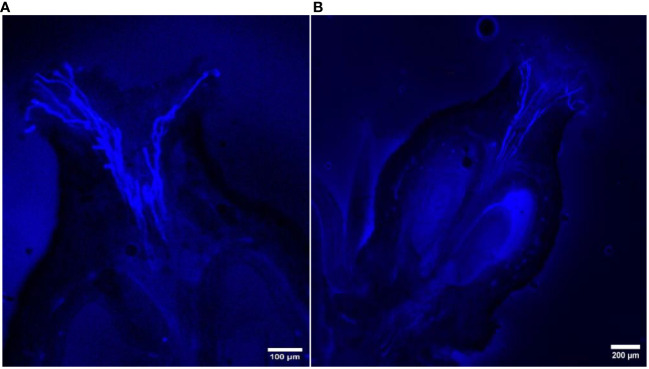
Longitudinal aniline blue-stained, fluorescent, frozen section (20 μm) of *Vitis parviflora* Roxb. × Male Hybrid (*V. vinifera* L.) flowers. Pollen germination on the stigma **(A)**. Pollen tubes reaching up to half of the stylar canal after 24 hours of pollination **(B)**.

**Figure 8:**
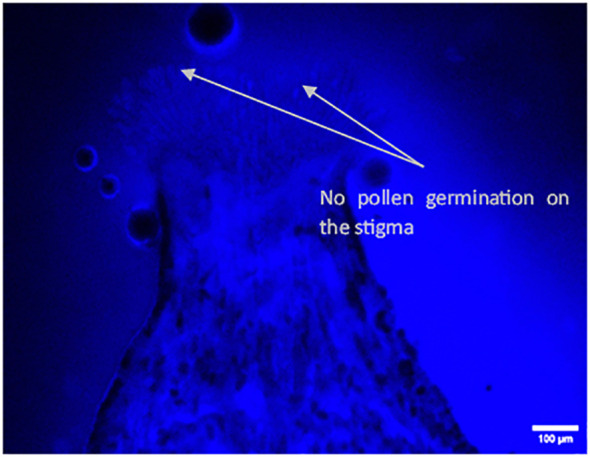
Longitudinal aniline blue-stained, fluorescent, frozen section (20 μm) of *Vitis parviflora* Roxb. × Dogridge (*Vitis champini* Planch.) flower. No pollen tube growth (pollen germination) on the stigma.

**Figure 9:**
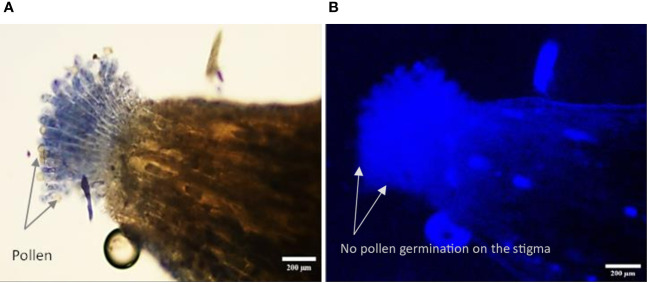
Longitudinal, frozen section (20 μm) of a selfed flower of *Vitis parviflora* Roxb. Bright-field image: papillous stigma, visible ungerminated pollen grains on the selfed *V. parviflora* Roxb. flower **(A)**. Fluorescent image showing no pollen tube growth (pollen germination) in the selfed flower of *V. parviflora* Roxb. **(B)**.

The *in vivo* study of pollen tube growth was conducted in three aspects, including pollen germination on the stigmatic surface and the initiation of pollen tube growth, the extension of pollen tubes reaching halfway or the base of the style canal, and the penetration of pollen tubes into the ovule. In VP/PN, notable pollen germination was observed on the stigmatic surface, leading to the abundant growth of pollen tubes within the stigma and style tissues (**Figure 5A**). Furthermore, there was significant growth of pollen tubes along the transmission tissue (**Figure 5B**). In the majority of the flowers, the pollen tubes grew significantly and extended to the base of the stylar canal. However, in a few flowers, the pollen tubes followed a shorter path, finishing their growth in the middle of the stylar canal. Notably, in some crossed flowers, the pollen tubes managed to penetrate the ovule by entering through the micropyle after 24 hours of pollination (**Figure 5C**). In VP/SC, good pollen germination was observed on the stigma along with profuse pollen tube growth in the stylar tissue (**Figure 6A**). Additionally, in most of the flowers, pollen tubes reached the base of the stylar canal after 24 hours of pollination. A significant number of pollen tubes along the transmission tissue were observed (**Figures 6B, C**). In VP/MH, slow pollen tube growth was observed, and pollen tubes entered more than half of the stylar canal after 24 hours of pollination but did not reach the base of the stylar canal (**Figures 7A, B**). In VP/DG, pollen grains did not germinate on the stigma (**Figure 8**). Similarly, in selfed flowers of VP, no pollen germination was observed on the stigmatic surface (**Figure 9**).

#### Crossability indices

Among the four crosses, the significantly higher berry retention percentage (61.14 %) was recorded in VP/SC followed by VP/PN (52.05 %) and the lowest (23.25%) in VP/MH (**Table 5**). No fruit set was observed in VP/DG. The compatibility index value for VP/SC was 1.183 while 1.154 for VP/PN. The lowest compatibility index (0.511) was recorded in VP/MH. The highest number of seeds per fruit was recorded in VP/PN (2.36) followed by VP/SC (2.246) and VP/MH (1.986).

**Table 5:** Fruit setting rate, average number of seeds per berry, and compatibility index of four interspecific crosses.

S. No.	Cross-combination	No. of pollinated flowers	Final berry retention (%)	Total seeds	Compatibility index	Average no. of seeds/berry
1.	VP/SC	792	61.14^a^	937	1.183	2.246^ab^
2.	VP/MH	654	23.25^b^	293	0.511	1.986^b^
3.	VP/PN	759	52.05^a^	876	1.154	2.36^a^
4.	VP/DG	782	0	0	0	0
	LSD (p ≤ 0.05)		(2.296)			(1.965)

Least significant difference (LSD) values in parentheses indicate LSD for transformed data.The same superscript indicates that the values do not differ significantly.

## Discussion

### Pollen micro-morphology

Palynology is primarily concerned with the study of various pollen features, which could be utilized in plant classification and phylogenetic studies (Claxton et al., 2005; Al-Hakimi et al., 2017) at the same time; the applied part of pollen-based information could be utilized for many purposes, *viz*., crop improvement and conservation of plant genetic resources. In this study, the basic purpose behind studying pollen-based features, i.e., the palynology of grape genotypes of three different species with asynchronous flowering (**Supplementary Figure 1**), was to find out the possibility of their utilization in successful pollination and fertilization for grape rootstock improvement. These three grape species, *V. vinifera* L., *V. champini* Planc., and *V. parviflora* Roxb., cannot be differentiated based on the pollen micro-morphology, as the genotypes that belonged to them did not indicate marked variation in parameters like polar, equatorial axes and colpus dimensions. Similar to our findings, Soares et al. (2013) observed variations in *Passiflora edulis* and *Passiflora setacea*, but these variations were not significant enough to differentiate them. However, Joujeh et al. (2019) found variations in pollen color, exine ornamentation pattern, and density of spines distributed on the exine surface of six species of *Centaurea* genus, which can be utilized for deciding taxonomic position.

Apertures are key pollen micro-morphology-based features for identifying pollen grains. According to Moore and Webb (1978), these apertures are the primary characteristics utilized in distinguishing variations among pollen grains or spore fossils. In this study, two categories of pollen were found based on the number of apertures. The first one comprises acolporated pollen found in DR (*V. champini* Planc.) and VP (*V. parviflora* Roxb.). The second group characterized by tricolporate pollen consists of eight grape genotypes, including genotypes from *V. vinifera* and *V. champini* Planc. The presence of an aperture determines the functionality of pollen. The presence of two forms of pollen (acolporate and tricolporate), i.e., pollen dimorphism, is documented in various species of *Vitis*, namely, *V. riparia* Michx. (Kevan et al., 1985), *Vitis aestivalis* Michx. (Kevan et al., 1988), and *Vitis coignetiae* Pulliat. (Kimura et al., 1988), and represents a form of functional dioecy. The presence of an aperture is also linked to the type of flower. Genotypes having inaperturate (acolporate) pollen typically exhibit well-developed pistils surrounded by reflexed and short stamens (Vasconcelos et al., 2009). This floral structure is linked with the production of inaperturate pollen, which is generally considered sterile or non-functional (Meneghetti et al., 2006; Barbieri et al., 2012). In contrast, tricolporate pollen is linked to hermaphrodite flowers (Pratt, 1971; Punt et al., 2003; Vasconcelos et al., 2009). Acolporate pollen lacks furrows (Furness, 2007); as a result, it cannot germinate (Abreu et al., 2006). Meanwhile, tricolporate pollen has three furrows, is fertile, and can germinate (Padureanu and Patras, 2018). This was further reflected in the fertility of the pollen of these genotypes (**Tables 3**, **4**; **Figures 5**–**9**). The presence of acolporate/inaperturate pollen within the same genus or species is linked with their different development from the normal one. A gene, INAPERTURATE POLLEN1 (INP1), specifically involved in the formation of pollen apertures, has been recently isolated and characterized in *Arabidopsis thaliana*; the loss of INP1 entails the lack of furrows and is responsible for the formation of inaperturate pollen (Dobritsa and Coerper, 2012). It further results in the uniform thickening of cell walls of pollen, lack of colpi, and an abnormal round shape. This could be the probable cause of acolporate pollen in DR and VP in this study.

Acolporated pollen in DR (*V. champini* Planc.) and *V. parviflora* Roxb. was unable to germinate on *in vitro* medium but was stained with TTC (**Figure 4**). The observed color change is attributed to the reduction of a colorless, soluble tetrazolium salt to form a visually detectable, reddish insoluble material known as formazan. This reduction is facilitated by dehydrogenase enzymes present specifically in metabolically active cells. However, there were no furrows on the pollen, so pollen tubes did not form (Caporali et al., 2003). A similar finding was reported in other species like *Solanum appendiculatum* (Zavada and Anderson, 1997) and *Arabidopsis* (Dobritsa and Coerper, 2012). Additionally, other morphological features, including shape and wall ornamentation, play a crucial role in pollen identification. However, dimension-based pollen parameters like polar axis, equatorial axis, mesocolpium width, and colpus length and width showed variation in different grape genotypes but did not follow any specific pattern with respect to the species they belong to (Lukšić et al., 2022). All the grape genotypes except EPS and BS exhibited foveolate-perforate exine ornamentation (Marasali et al., 2005; Abreu et al., 2006; Lukšić et al., 2022). EPS and BS both are from *V. vinifera* L., and they showed reticulate (Padureanu and Patras, 2018) and reticulate striate exine ornamentation, respectively.

### Pollen storage

Fresh pollen of each grape genotype exhibited a distinct pollen germination percentage. For instance, FS recorded a germination rate of 90.4 %, while POC showed a lower rate of 40.4 %. This suggests that the pollen from each genotype possesses inherent characteristics influencing its ability to germinate under specific conditions as observed by Kelen and Demirtas (2003) in grapes. Similarly, significant variation in fresh pollen germination has been reported within the same genus or species, as reported in the case of the Serbian Autochthon Apple (Ćalić et al., 2021) and crape myrtle (*Lagerstromia* spp.) (Akond et al., 2012). Probably, this could be a reason behind the fact that some grape genotypes are good pollinators as compared to others (Pereira et al., 2018; Wang et al., 2022), similar findings have been reported in Annona (Pereira et al., 2014) and plum (Đorđević et al., 2022).

The effect of different storage temperatures and durations on *in vitro* pollen germination revealed that the initial pollen germination was the highest on the day of collection for all genotypes and reduced as storage duration was prolonged. Storage of pollen at lower temperatures (4 °C, −20° C, and −80 °C) has been reported to increase its longevity in various plant species, as reported in *Pistachio* (Aldahadha et al., 2020), hazel (Novara et al., 2017), mango (Dutta et al., 2013), litchi (Wang et al., 2015), and kiwi (Borghezan et al., 2011). The reduction in pollen germination percentage was significant at room temperature but minimal at −196 °C. As pollen ages, the following occurs: a deterioration in its intracellular structure, a decline in enzyme activity, an increase in the presence of free radicals, and processes such as de-esterification and lipid peroxidation. These factors collectively result in increased cellular component leakage when the pollen is rehydrated (Taylor and Hepler, 1997). When pollen is stored at low temperatures, the metabolic processes within the pollen grains may be diminished or suppressed, leading to an extension of the pollen grain lifespan (Ćalić et al., 2021; Đorđević et al., 2022). This phenomenon explains the minimal alterations in pollen germination and viability observed throughout the entire storage duration at −196 °C. In the study, the logic behind the selection of these four temperatures was to check the possibility of successful pollen storage under available storage facilities to support the grape hybridization program. If pollen needs to be stored for next-day pollination use, it can be stored at room temperature. For a 30-day storage period, pollen can be effectively stored at 4 °C for each genotype, excluding POC. Laboratories commonly have refrigerators, making this a practical option. However, for genotypes involved in grape hybridization and with access to storage facilities at −20 °C and −196 °C (liquid nitrogen), it is advisable to utilize these options for optimal pollen storage within the 30-day timeframe of the flowering period (**Supplementary Figure 1**). This ensures successful pollen preservation for use in the grape hybridization program.

### Male sterility: an indicator of functional femaleness

Our findings indicated that VP and DR had acolporated pollen grains lacking apertures (**Table 1**), preventing germination on *in vitro* pollen germination medi (Anderson and Symon, 1989; Penny and Steven, 2009). Similarly, self-pollination experiments with VP and VP/DG confirmed the absence of pollen germination on the stigma (**Figures 8**, **9**). This proves that VP and DR are male sterile or functional female and can be utilized as female parents without the need for emasculation, thus saving time and effort (Kaul, 1988).

### Cross-compatibility and pollen–pistil interaction

Four cross-combinations belonged to *V. parviflora* Roxb. × *V. vinifera* L. (VP/MH and VP/PN), *V. parviflora* Roxb. × *V. champini* Planc. (VP/SC and VP/DG), and *V. parviflora* Roxb. × *V. parviflora* Roxb. VP/VP was studied to find out cross-compatibility (**Figures 5**–**9**). It was evident from the pollen tube growth inside the pistil in VP/SC and VP/PN that it had a high degree of compatibility in pollen–pistil interaction. Aniline blue fluorescence microscopy revealed a greater number of germinating pollen on the stigmatic surface, with pollen tubes reaching the base of the stylar canal and even entering the ovules through the micropyle within 24 hours of pollination in VP/SC and VP/PN combinations. This strong compatibility was reflected in higher fruit set percentages, an increased average number of seeds per berry, and compatibility indices in VP/SC and VP/PN cross-combinations. More pollen tube growth (García-Breijo et al., 2020) and more seeds per berry indicate more compatibility among parents (Békefi and Halász, 2005; Cerovic et al., 2021).

While in VP/MH, pollen tubes did not reach the base of the stylar canal; most of the pollen tubes reached more than half of the stylar canal after 24 hours of pollination. Despite that, slower pollen tube growth for successful fertilization occurred, resulting in the production of seeds. Thus, the pollen tubes must have continued to grow for more than 24 hours after pollination and then fertilized the ovules. As a result, lower berry retention (23.25%) and number of seeds per berry (1.986) were recorded. The slower pollen tube growth could have led to reduced fertilization and subsequent fruit set as reported by Adachi et al. (2009) in autotetraploid cultivars of apple. Similarly, Pasonen et al. (2001) reported a significant positive correlation between pollen-tube growth rate and seed mass in *Betula pendula*. Conversely in VP/MH, although the *in vitro* pollen germination was higher (71.7 %) (*at par* with SC), the fruit set (23.25 %) was significantly less as compared to VP/SC along with the lowest compatibility index (0.511) among all successful cross-combinations (Badiger et al., 2023).

## Conclusion

The present study revealed diverse palynological characteristics based on the presence or absence of colpus and variations in pollen dimensions. There was no particular trend for these parameters with respect to the species. The study also identified the presence of male sterility in *V. parviflora* Roxb. and Dogridge. The pollen storage temperatures −20 °C and −196 °C were found to be efficient for up to 30 days. *V. parviflora* Roxb. × *V. vinifera* L. (Pusa Navrang) and *V. parviflora* Roxb. × *V. champini* Planc. (Salt Creek) stood out as highly cross-compatible combinations, showing superior fertility indices, and may be exploited for the rootstock breeding of grapes.

## Data availability statement

The original contributions presented in the study are included in the article/**Supplementary Material**, further inquiries can be directed to the corresponding author/s.

## Author contributions

PR: Conceptualization, Data curation, Formal analysis, Funding acquisition, Investigation, Methodology, Writing – original draft, Writing – review & editing. MT: Conceptualization, Data curation, Formal analysis, Methodology, Writing – original draft, Writing – review & editing, Resources. MV: Conceptualization, Methodology, Writing – review & editing. CK: Investigation, Methodology, Writing – review & editing. JP: Methodology, Writing – review & editing. VS: Methodology, Writing – review & editing. SP: Methodology, Writing – review & editing. NM: Methodology, Writing – review & editing. GC: Methodology, Writing – review & editing. PM: Methodology, Writing – review & editing. HK: Methodology, Writing – review & editing. AJ: Writing – review & editing. EV: Data curation, Writing – review & editing. VP: Resources, Writing – review & editing. SS: Resources, Writing – review & editing.
