# Supplementary figures and images for: Studies on pollen micro-morphology, pollen storage methods, and cross-compatibility among grape (Vitis spp.) genotypes
Source: Front Plant Sci. 2024 Feb 21;15:1353808. doi: 10.3389/fpls.2024.1353808 (PMC10922203; doi:10.3389/fpls.2024.1353808)

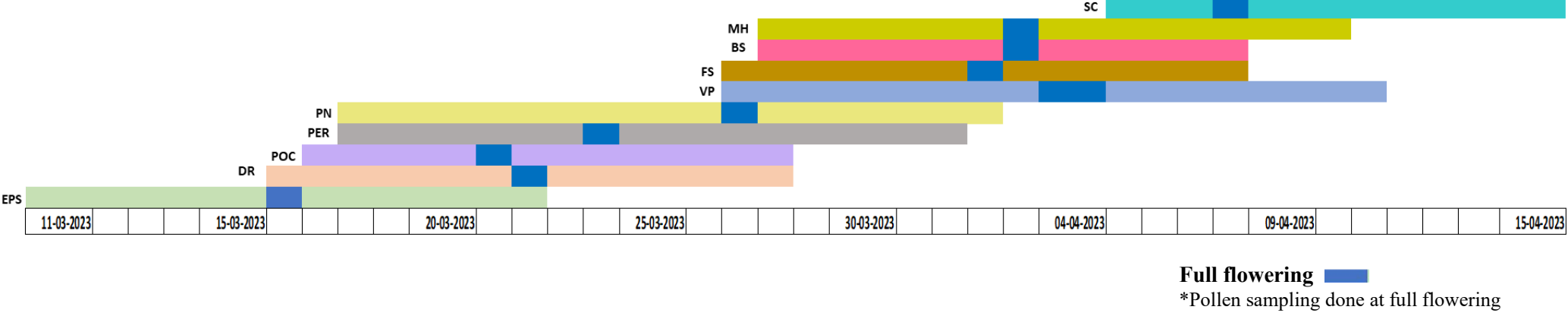

**SUPPLEMENTARY FIGURE 1**| Flowering period of grapes (*Vitis* spp.) genotypes in the year 2023

Supplement: Supplementary file 1 [file Image_1.pdf]
