# Supplementary material for: Studies on pollen micro-morphology, pollen storage methods, and cross-compatibility among grape (Vitis spp.) genotypes
Source: Front Plant Sci. 2024 Feb 21;15:1353808. doi: 10.3389/fpls.2024.1353808 (PMC10922203; doi:10.3389/fpls.2024.1353808)
Supplement: Supplementary file 2 [file Table_1.docx]

**SUPPLEMENTARY TABLE**| Standardization of *in vitro* pollen germination media for different grape genotypes

| S No. | Media | Pollen germination (%) among different media compositions | | | | | | | |
| --- | --- | --- | --- | --- | --- | --- | --- | --- | --- |
|  |  | EPS | PN | POC | PER | BS | FS | MH | SC |
|  | M1 | 24.8^d^ | 21.67^c^ | 14.05^e^ | 19.84^d^ | 25.7^d^ | 35.67^e^ | 25.94^d^ | 33.31^e^ |
|  | M2 | 19.55^d^ | 24.32^c^ | 18.66d^e^ | 21.59^d^ | 30.84^d^ | 31.33^e^ | 24.93^d^ | 30.42^e^ |
|  | M3 | 38.5^c^ | 36.24^b^ | 22.44^cd^ | 35.62^c^ | 41.34^c^ | 65.24^c^ | 48.65^b^ | 55.47^c^ |
|  | M4 | 71.66^a^ | 61.66^a^ | 41.06^a^ | 65.17^a^ | 68.97^a^ | 90.74^a^ | 72.37^a^ | 77.03^a^ |
|  | M5 | 24.8^d^ | 31.45^b^ | 28.45^bc^ | 29.4^c^ | 38.94^c^ | 46.24^d^ | 34.59^c^ | 49.86^d^ |
|  | M6 | 54.03^b^ | 55.94^a^ | 34.41^ab^ | 53.98^b^ | 59.64^b^ | 84.27^b^ | 68.45^a^ | 72.64^b^ |
|  | LSD | (3.34) | (2.70) | (3.24) | (2.67) | (2.55) | (2.54) | (2.14) | (1.65) |

#LSD values in parenthesis indicate LSD for transformed data.

M1:10% sucrose, M2:20% sucrose, M3:10 % sucrose+100 mg/l boric acid, M4:10 % sucrose+100 mg/l boric acid+300 mg/l calcium nitrate, M5:20 % sucrose+100 mg/l boric acid, M6:20 % sucrose+100 mg/l boric acid+300 mg/l calcium nitrate
